# Adrenocortical Adenoma With Protrusion Into the Inferior Vena Cava Initially Suspected to Be Adrenocortical Carcinoma

**DOI:** 10.1210/jcemcr/luae043

**Published:** 2024-04-23

**Authors:** Hiroki Takizawa, Reo Higuchi, Yuki Fukumura, Muga Terasawa, Katsuhiro Sano, Hiromasa Goto

**Affiliations:** Department of Metabolism & Endocrinology, Juntendo University Graduate School, Tokyo 113-8421, Japan; Department of Radiology, Juntendo University Graduate School of Medicine, Tokyo 113-8421, Japan; Department of Human Pathology, Juntendo University Graduate School of Medicine, Tokyo 113-8421, Japan; Department of Hepatobiliary-Pancreatic Surgery, Juntendo University School of Medicine, Tokyo 113-8421, Japan; Department of Radiology, Juntendo University Graduate School of Medicine, Tokyo 113-8421, Japan; Department of Metabolism & Endocrinology, Juntendo University Graduate School, Tokyo 113-8421, Japan

**Keywords:** adrenocortical adenoma, protrusion into the inferior vena cava

## Abstract

Adrenal tumors with invasion into the inferior vena cava (IVC) are typically malignant. Here, we present a case of adrenocortical adenoma with protrusion into the IVC. A 70-year-old man was referred to our hospital after his magnetic resonance imaging scan of the abdomen coincidently revealed a right adrenal tumor invading the IVC. We suspected an aggressive adrenal carcinoma and tumor resection was performed. However, all 3 existing pathological criteria (Weiss, modified Weiss, and Helsinki) suggested the tumor was benign. Immunohistochemistry for CD31 showed the tumor inside the central adrenal vein (CAV), right adrenal vein (RAV), and IVC was entirely covered with CD31-positive vascular endothelial cells. The CAV is known to sometimes lack smooth muscle in its walls and normal adrenocortical cells covered by endothelial cells sometimes protrude into the CAV from this gap. These findings suggest that this tumor likely protruded into the IVC by pushing against the CAV wall, rather than by invasion into the vascular wall. In the case with adrenal tumors protruding into the IVC, the fact that the tumor surface was covered by vascular endothelial cells was considered supportive of its benign nature.

## Introduction

Endocrinologists usually consider adrenal tumors with extension into the inferior vena cava (IVC) to be high-grade malignancies, such as adrenocortical carcinoma (ACC), pheochromocytoma, or metastatic tumors (1). Among these 3 malignancies, ACC is the most common; urgent surgical resection is necessary due to poor prognosis. Here, we describe a case of adrenocortical adenoma (ACA) with extension into the IVC for which we could prove the benign nature of the tumor. This is a report of a rare case of ACA with protrusion into the IVC.

## Case Presentation

A 70-year-old Japanese man was referred to our hospital after his magnetic resonance imaging (MRI) scan to evaluate his abdominal pain revealed a right adrenal tumor with suspected invasion into the IVC. At the first visit, he complained of no particular symptoms. He has hypertension and paroxysmal atrial fibrillation and is being treated with losartan potassium 25 mg and rivaroxaban 15 mg, but he has no history of paroxysmal hypertension. The patient's height was 170 cm, weight was 85 kg, and blood pressure was 124/58 mmHg. He was obese by Asian criteria (body mass index 29.4 kg/m^2^), but he did not have any symptoms consistent with Cushing syndrome.

## Diagnostic Assessment

**Figure 1.**
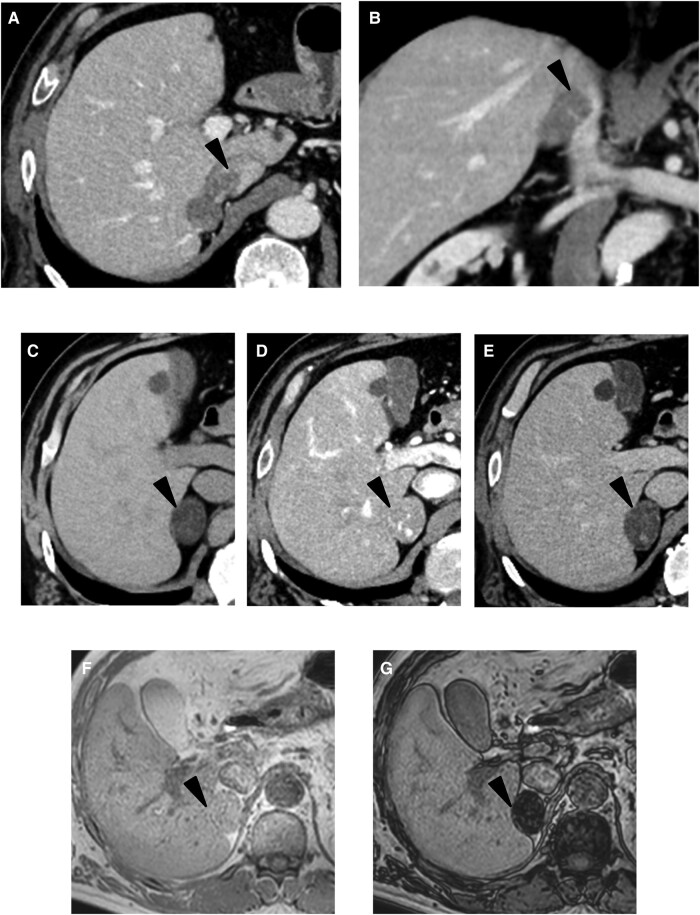
Computed tomography (CT) and magnetic resonance imaging (MRI) findings. Contrast-enhanced abdominal CT in axial (A) and coronal (B) reveals a tumor originating from the right adrenal gland extending into the IVC via the right adrenal vein (black arrowhead). Non-contrast imaging (C) showed low attenuation, intense enhancement in the arterial phase (D), and washout in the delayed phase (E). On T1-weighted MRI, there was a distinct signal reduction in the opposed phase (G) when compared to the in phase (F). The adrenal gland tumor is indicated by a black arrowhead.

Contrast-enhanced computed tomography (CT) was performed to confirm further morphological changes in the adrenal gland, which revealed a well-defined 35-mm mass in the right adrenal gland. The mass had a smooth edge, displayed internal homogeneity, and extended into the IVC, forming a tumor invasion greater than 19 mm (Fig. 1A and 1B). The mass had an average CT value of 3 Hounsfield units on unenhanced CT. On enhanced CT, marked enhancement of the tumor with contrast administration during the arterial phase with washout in the delayed phase was observed (Fig. 1C-1E). The measurement by contrast-enhanced CT revealed an absolute percentage washout of 63.6% and a relative percentage washout of 61.3%. Re-evaluation of the MRI findings showed a clear signal reduction in the opposed phase compared to the in phase on T1-weighted chemical shift images (Fig. 1F and 1G).

Laboratory data, including complete blood cell count, coagulation tests, hepatic, renal, and lipid profiles, were within normal reference ranges. In addition, his fasting blood glucose and glycated hemoglobin (HbA1c) were 92 mg/dL (5.1 mmol/L) and 5.8% (normal reference range, 4.6%-6.2%), respectively. Endocrinologic blood testing (Table 1) showed plasma adrenocorticotropic hormone (ACTH) of 22.1 pg/mL (4.86 pmol/L) (normal reference range, 10-60 pg/mL; 2.20-1302 nmol/L) and serum cortisol levels of 13.5 μg/dL (372 nmol/L) (normal reference range, 6.0-18.4 μg/mL; 165.5-50.7.7 nmol/L) in the early morning (8:00 Am) and < 3 pg/mL (< 0.6 pmol/L) and 2.3 μg/dL (63.4 pmol/L) during late night (11:00 Pm), respectively. The circadian rhythm of ACTH and cortisol was maintained. On the other hand, the serum cortisol level during a 1-mg overnight dexamethasone suppression test was 8.9 μg/dL (245 nmol/L), indicating no suppression. Furthermore, 24-hour urinary free cortisol excretion was as high as 198 μg/day (546 nmol/day) (normal reference range, 5.5-66.7 μg/day; 15-184 nmol/L). The levels of aldosterone (measured by radioimmunoassay) were 2.26 ng/dL (62.6 nmol/L) (normal reference range, 3.0-16.0 ng/dL; 83.1-443.2 pmol/L), and plasma renin activity was 0.6 ng/mL/hour (0.6 μg/L/hour) (normal reference range, 0.3-2.9 ng/mL/hour; 0.3-2.9 μg/L/hour). The aldosterone-renin activity ratio was 3.7, indicating that the presence of primary aldosteronism is unlikely. Additionally, the plasma metanephrine was 15 pg/dL (78.9 nmol/L) (normal reference range, 0-130 pg/dL; 0-683.8 pmol/L), and plasma normetanephrine was 35 pg/dL (191.1 nmol/L) (normal reference range, 0-506 pg/dL; 0-2762.8 pmol/L). These findings suggest that the presence of pheochromocytoma is also unlikely.

**Table 1.** Endocrinological blood test results

Blood	Result	Normal reference range
ACTH	22.1 pg/mL (4.86 pmol/L)	10-60 pg/mL (2.20-13.2 pmol/L)
Cortisol	13.5 μg/dL (372 nmol/L)	6.0-18.4 μg/dL (165.5-507.7 nmol/L)
Testosterone	5.03 ng/mL (17.4 nmol/L)	1.92-8.84 ng/mL (8.66-38.1 nmol/L)
DHEA-S	24 μg/dL (0.70 μmol/L)	24-244 μg/dL (0. 70-6.60 μmol/L)
Aldosterone (RIA)	2.26 ng/dL (62.6 pmol/L)	3.0-16.0 ng/dL (83.1-443.2 pmol/L)
Plasma renin activity	0.6 ng/mL/hour (0.6 μg/L/hour)	0.3-2.9 ng/mL/hour (0.3-2.9 μg/L/hour)
Metanephrine	15 pg/mL (78.9 pmol/L)	0-130 pg/mL (0-683.8 pmol/L)
Normetanephrine	35 pg/mL (191.1 pg/mL)	0-506 pg/mL (0-2762.8 pmol/L)
Urine		
Free cortisol	198 μg/day (546 nmol/day)	5.5-66.7 μg/day (15-184 nmol/day)
Metanephrine	0.14 mg/day (7.1 μmol/day)	0.1-0.28 mg/day (2.8-14.7 μmol/day)
Normetanephrine	0.22 mg/day (12.0 μmol/day)	0.15-0.41 mg/day (8.2-22.4 μmol/day)
Androsterone	0.97 mg/day (3.34 μmol/day)	1.10-4.20 mg/day (3.78-14.4 μmol/day)

Abnormal values are shown in bold font. Values in parenthesis are International System of Units (SI).

Abbreviations: ACTH, adrenocorticotropic hormone; DHEA-S, dehydroepiandrosterone sulfate; RIA, radioimmunoassay.

Based on these findings, we made a preoperative diagnosis of ACC with autonomous cortisol production.

## Treatment

**Figure 2.**
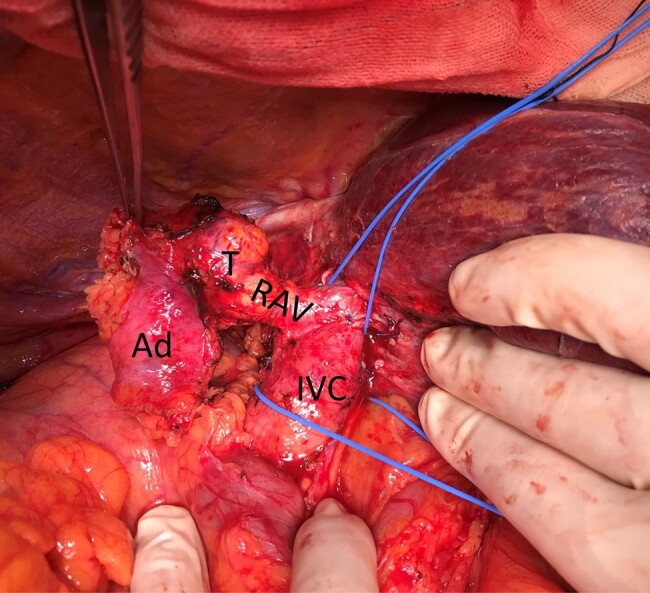
Intraoperative findings. The tumor (T) originated from the right adrenal gland (Ad) and extended into the inferior vena cava (IVC) via the right adrenal vein (RAV).

Based on the preoperative diagnosis of ACC, the patient underwent laparotomy to ensure complete removal of the primary tumor and to minimize the risk of intraoperative seeding. Laparotomy was performed through an inverted L-shaped incision. No intra-abdominal dissemination was observed on visual inspection, which was confirmed with negative ascites cytology. After assessing the extent of tumor protrusion into the IVC using intraoperative ultrasonography, excision of the protruding tumor involved clamping the IVC with vascular forceps (Fig. 2). Surprisingly, the tumor easily slipped out of the IVC. The IVC defect was closed using a patch made of Gerota's fascia. Complete tumor resection was achieved.

Perioperative steroid replacement therapy was started based on the release of autonomous cortisol production and invasive stress. On postoperative day 6, a cosyntropin stimulation test was conducted, which revealed decreased cortisol responsiveness (Table 2). Consequently, the patient required continuous hydrocortisone replacement therapy at a dose of 15 mg per day.

**Table 2.** Cosyntropin stimulation test results before or after surgery

Serum cortisol level	0 minutes	30 minutes	60 minutes
Before surgery	13.5 μg/dL (372 nmol/L)	29.7 μg/dL (819 nmol/L)	38.5 μg/dL (1062 nmol/L)
After surgery	4.4 μg/dL (121 nmol/L)	5.7 μg/dL (157 nmol/L)	6.2 μg/dL (171 nmol/L)

**Figure 3.**
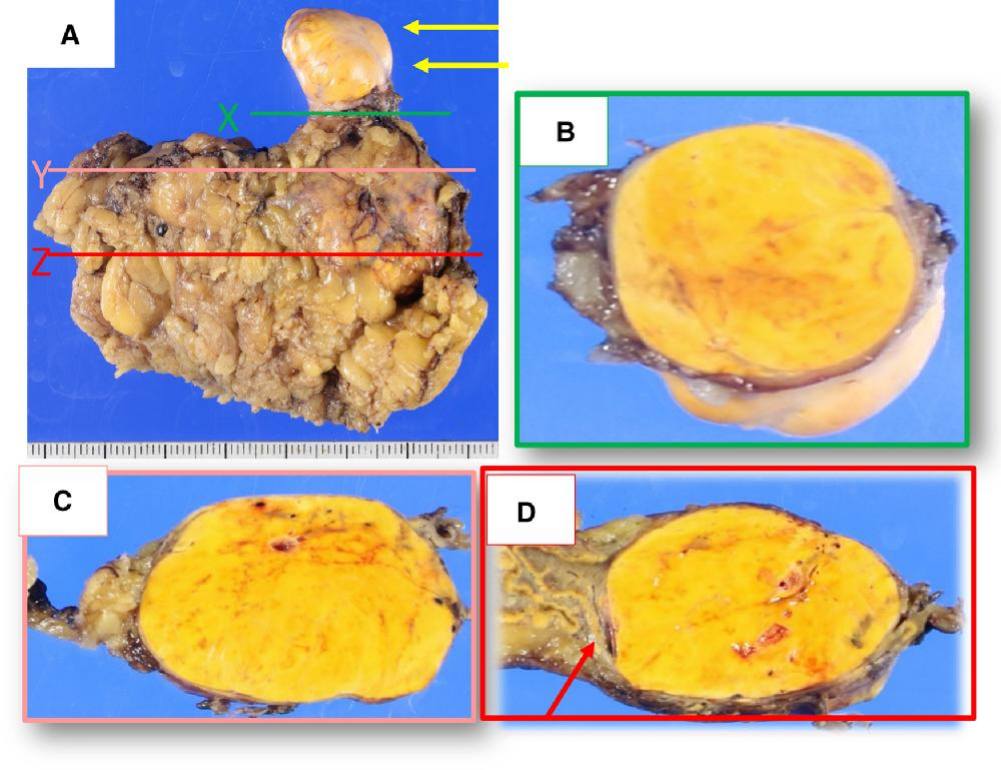
Adrenal tumor gross examination. (A) Grossly, there was a yellowish tumor in the right adrenal gland that extended into adrenal vein and inferior vena cava (IVC) (yellow arrows show the tumor protruding from the IVC.). (B) Cut section at level X in Fig. 3A showed that the IVC was filled with yellowish tumor. (C) Cut section at level Y and (D) Cut section at level Z in Fig. 3A. The central adrenal vein was grossly evident in (D) (red arrow), whereas it was not recognizable in (C).

Grossly, a well-circumscribed, yellowish tumor (26 × 22 × 48 mm) was seen in the right adrenal gland. It protruded into the right adrenal vein (RAV) and IVC (Fig. 3A and B). The central adrenal vein (CAV) was compressed and slightly dilated in cut section Z and not seen in cut section Y (Fig. 3C and 3D).

**Figure 4.**
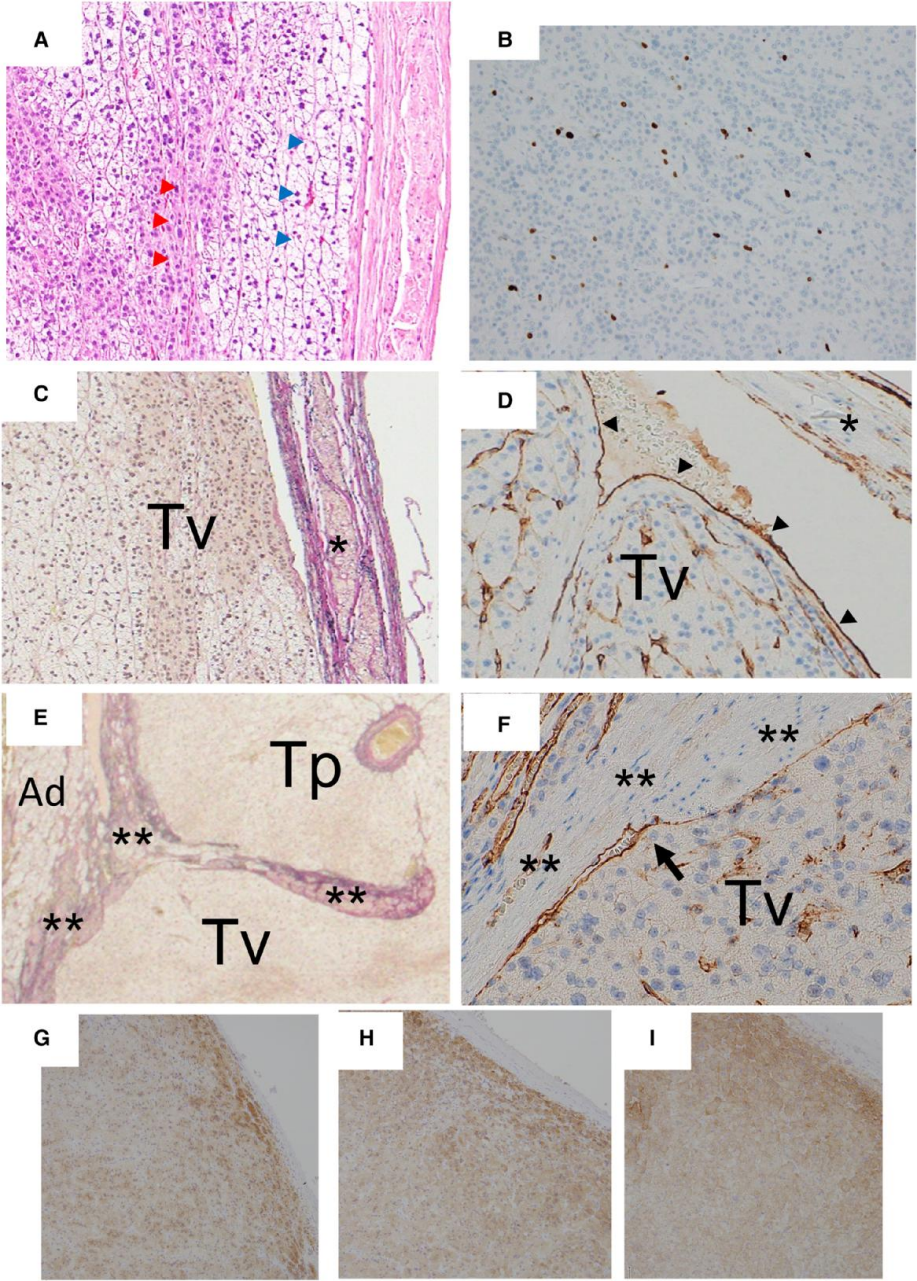
Macroscopic morphology of the adrenal tumor. (A) Histologically, the tumor predominantly consisted of clear cells mimicking the cells of the zona fasciculata (blue arrowhead) as well as eosinophil cells mimicking the cells of the zona reticularis (red arrowhead). (B) The Ki-67 labeling index was approximately 5% (immunohistochemistry for MIB-1). (C, D) Microscopic view of the cut section X in Fig. 3A shows tumor (Tv) in the IVC (C: Elastica van Gieson stain). Arrowheads show the tumor surface was covered with endothelial cells (D: immunohistochemistry for CD31). * Indicates the IVC wall with smooth muscle. (E, F) Microscopic view of the cut section Y in Fig. 3A shows tumor growing inside the adrenal parenchyma (Tp) and inside the central adrenal vein (Tv) (E: Elastica van Gieson stain). The bold arrow shows that the tumor surface inside the central adrenal vein was covered with endothelial cells (F: immunohistochemistry for CD31). ** indicates the wall of the central adrenal vein with smooth muscle. (G-I) Tumor cells were positive for steroid 17α-hydroxylase (G), 3β-hydroxysteroid dehydrogenase (H), and steroid 11β-hydroxylase (I).

Histologically, the tumor grew in a cord-like or alveolar-like pattern and lacked a diffuse proliferative component. The tumor was predominantly composed of cells with foamy cytoplasm, resembling the normal adrenal cortical fascicular layers (80%-90%), along with oxyphilic cells (10%-20%) (Fig. 4A). Mild nuclear irregularities and enlargement were observed in tumor cells. However, severe atypia and abnormal mitotic figures were not seen. No necrotic foci, capsular invasion, or sinusoidal invasion was observed. The Ki-67 labeling index was approximately 5% (Fig. 4B). Hence, the tumor had scores of 0, 0, 5 according to the Weiss criteria (2), modified Weiss criteria (3), and Helsinki criteria (4), suggesting that the tumor was benign. Thus, we diagnosed this case as ACA. Since protrusion of adrenocortical cells into the CAV through the thin part of vein wall was present, immunohistochemistry for CD31 (platelet endothelial cell adhesion molecule-1, a vascular endothelial cell marker) (Fig. 4C-4F) was performed. It showed that the tumor in the RAV and IVC was entirely covered with CD31-positive endothelial cells.

Based on this immunohistochemistry finding, the mechanism of tumor extension into the RAV and IVC was shown to be protrusion (extension into the vessel cavity by pushing or compressing the vessel wall), not vessel invasion (extension into the vessel cavity via destruction of the vessel wall) (Fig. 4C and 4D). Furthermore, with detailed histological examination, the tumor entrance site into the CAV was detected. The intra-CAV tumor was covered with CD31-positive endothelial cells as well (Fig. 3A, section Y and Fig. 4E and 4F). In addition, tumor was immunopositive for steroid 17α-hydroxylase (Fig. 4G), 3β-hydroxysteroid dehydrogenase (Fig. 4H), and steroid 11β-hydroxylase (Fig. 4I).

## Outcome and Follow-Up

During the postoperative clinical course, we discontinued hydrocortisone supplementation 9 months after surgery. The patient maintained normal adrenal function at 14 months after surgery (serum ACTH, 125 pg/mL [27.5 pmol/L]; cortisol, 13.1 μg/dL [364 nmol/L]). At 1 year after surgery, no tumor recurrence or metastasis had been detected.

## Discussion

A learning point from this case is that ACAs can also protrude into the IVC. Chesson et al reported that among 105 adrenal tumors with protrusion into the IVC, 78 were diagnosed as ACC, 16 as pheochromocytoma, 3 as neuroblastoma, 3 as leiomyosarcoma, 2 as transitional epithelial carcinoma, 2 as metastatic small cell carcinoma, and 1 as Wilms’ tumor (1). Furthermore, 74% of these tumors were right-sided adrenal tumors and ACA has not been reported as a contributing cause of tumor with protrusion into the IVC yet. In retrospect, the CT and MRI imaging findings were consistent with ACA, such as the presence of fat content and contrast washout. Consequently, the radiologist diagnosed this tumor as an atypical ACC. However, based on previous reports, we had no doubt about the malignancy, and we believed the best treatment for this patient was tumor resection. We think one of the novelties of this case report is that we pathologically proved that the entire tumor surface in the IVC and RAV was covered with CD31-positive endothelial cells, which had not been previously described for ACC in any textbooks or previous reports. Regarding the origin of these CD31-positive cells, we consider endothelial cells from the CAV as the most likely source. CAV is a muscular vein that has thick smooth muscle in its media in most parts. However, it sometimes lacks smooth muscle where tributaries enter (5). Normal adrenal cortex, which is covered by only vascular endothelial cells, sometimes protrudes into the CAV from that thin part. With this pathological feature as well as other features in the Weiss, modified Weiss, and Helsinki criteria, the tumor was diagnosed as benign ACA rather than ACC.

We have still some concerns about the pathological differentiation between ACC and ACA. The Weiss criteria, proposed in 1984, comprises 9 pathological findings. They are sometimes difficult to use; in particular, nuclear dysmorphism and vascular invasion are sometimes difficult to determine. We were also concerned about sampling error and observer variation, as demonstrated by a case report of ACC with metastasis 4 to 5 years after the diagnosis of ACA (6). In recent years, attempts have been made to enhance the diagnostic accuracy of the modified Weiss score (3) and the Helsinki score (4) by incorporating immunohistochemical evaluations, including the Ki-67 labeling index. We attempted to reduce diagnostic limitations by having readings by 2 independent endocrine pathologists and using 3 different criteria. The patient is cured but needs long-term follow-up.

For primary adrenal tumors with protrusion into the IVC that are radiologically and pathologically suspected to be benign, confirmation of CD31-positive endothelial cells on the tumor surface might be helpful for determining the nature of the adrenal tumor.

## Learning Points

- ACA can protrude into the IVC.
- ACA with tumor protrusion into the IVC can be demonstrated radiologically and pathologically.
- CD31-positive endothelial cells on the surface of an intravascular tumor might help determine its nature.

## Data Availability

Original data generated and analyzed during this study are included in this published article.

## Abbreviations

ACA: adrenocortical adenoma
ACC: adrenocortical carcinoma
ACTH: adrenocorticotropic hormone
CAV: central adrenal vein
CT: computed tomography
IVC: inferior vena cava
MRI: magnetic resonance imaging
RAV: right adrenal vein
